# Gerbode defect following endocarditis and misinterpreted as severe pulmonary arterial hypertension

**DOI:** 10.1186/1476-7120-8-44

**Published:** 2010-09-30

**Authors:** Nereida Xhabija, Edvin Prifti, Iris Allajbeu, Fatmir Sula

**Affiliations:** 1 Department of Cardiology and Cardiac-Surgery, American Hospital, Department of Cardiology, Tirana, Albania; 2 Department of Radiology, American Hospital, Department of Cardiology, Tirana, Albania

## Abstract

A Gerbode -type defect is a ventricular septal defect communicating directly between the left ventricle and right atrium. It is usually congenital, but rarely is acquired, as a complication of endocarditis. This can be anatomically possible because the normal tricuspid valve is more apically displaced than the mitral valve. However, identification of an actual communication is often extremely difficult, so a careful and meticulous echocardiogram should be done in order to prevent echocardiographic misinterpretation of this defect as pulmonary arterial hypertension. The large systolic pressure gradient between the left ventricle and the right atrium would expectedly result in a high velocity systolic Doppler flow signal in right atrium and it can be sometimes mistakably diagnosed as tricuspid regurgitant jet simulating pulmonary arterial hypertension.

We present a rare case of young woman, with endocarditis who presented with severe pulmonary arterial hypertension. The preoperative diagnosis of left ventricle to right atrial communication (acquired Gerbode defect) was suspected initially by echocardiogram and confirmed at the time of the surgery.

A point of interest, apart from the diagnostic problem, was the explanation for its mechanism and presentation. The probability of a bacterial etiology of the defect is high in this case.

## Introduction

Gerbode's defect is a rare form of VSD that allows for communication between the LV and RA. Two types of LV-to-RA communication are described: supravalvular and infravalvular, depending on whether the defect in the membranous septum is above or below the tricuspid valve [1,2]. Although the majority appears to attribute the Gerbode defect to congenital origins, there have been publication in the past that have credited the defect's formation secondary to bacterial endocarditis, especially supravalvular form [3].

We present a case of our patient with this uncommon complication of endocarditis, simulating severe pulmonary hypertension.

## Case report

**Figure 1:**
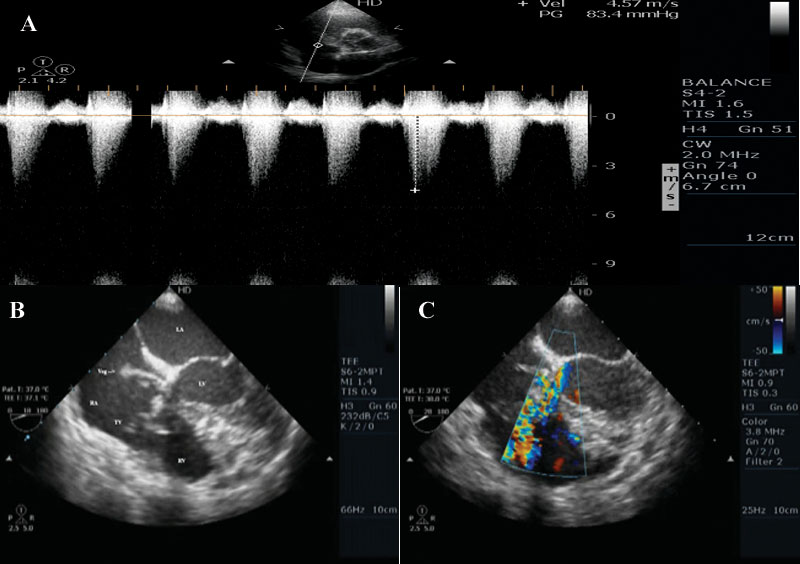
**High velocity jet from Gerbode type defect mixed with tricuspid regurgitation jet. RV, right ventricle; LV, left ventricle; RA, right atrium; LA, left atrium;TV, tricuspid valve; Veg, vegetation. **A) Continuous wave Doppler from Gerbode type defect mixed with tricuspid regurgitation jet revealing a high velocity jet simulating severe pulmonary arterial hypertension with estimated systolic pulmonary arterial pressure of 83 mmHg. B) Transeosophageal echocardiography four-chamber view demonstrating a large vegetation in RA located just above the tricuspid valve septal leaflet. Its attachment was not precisely defined, but no obstruction was identified at the level of tricuspid valve. C) The color Doppler demonstrating a systolic flow between the LV and both RV and RA.

A 41 year old lady, from Kosovo, was referred to our hospital for severe pulmonary arterial hypertension and a mass in right atrium suspected for vegetation. About one month before, she presented to the ER of her local hospital with pyrexia and malaise. She was given medication and nutrition through the right internal jugular site for a few days since her peripheral venous system was unavailable. Cardiology was consulted because her blood culture grew methacilin sensitive Staphylococcus aureus and she had persistent fever, despite antibiotics treatment. At that time the echocardiogram was interpreted as following: normal valves and cardiac chambers, but a mobile mass consistent with vegetation in right atrium was present. The tricuspid regurgitation was considered mild- to- moderate with estimated pulmonary arterial systolic pressure about 80-90 mmHg (figure 1A).

At the time of her presentation she had a 40 grade fever and complained of fatigue and palpitation. A 3/6 systolic murmur over the left precordium was audible, while pulmonary auscultation was negative. Her C-reactive protein, white cell count and erythrocyte sedimentation rate were elevated, Hb level was 9.6 and D-dimer was very high (3088 ng/ml). An electrocardiogram revealed sinus tachycardia with right bundle branch block.

**Figure 2:**
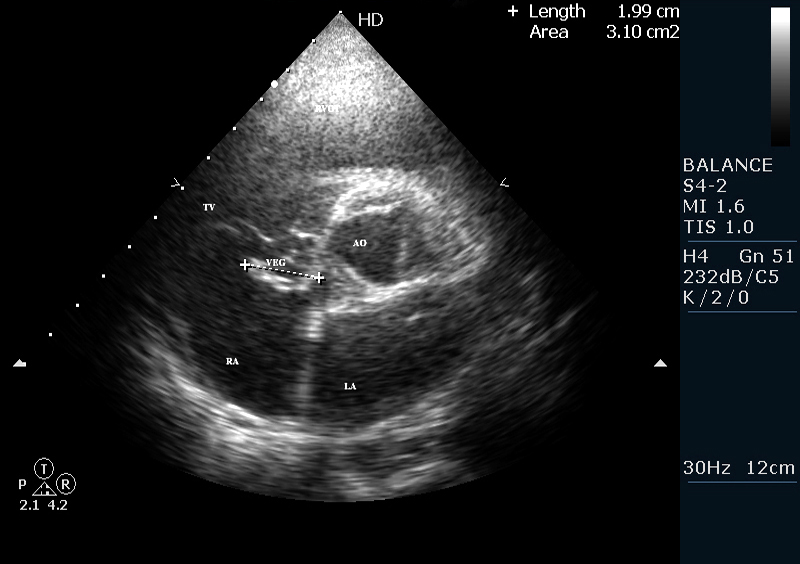
**Transthoracic short axis parasternal view measuring the vegetation about 20 mm long**.

The patient came to us for further investigation and an echocardiogram was repeated at our institution and reviewed in detail. It demonstrated normal valves and cardiac chambers, but also demonstrated a large, irregularly shaped, oscillating and highly mobile vegetation in the RA located above to the tricuspid valve septal leaflet (figure 1B and 2). Its attachment was not precisely seen, but the mass did not cause any obstruction at the level of tricuspid valve (Additional file 1: video S1; and Additional file 2: Video S2).

On closer inspection and careful review of her echocardiogram, we visualized a clear jet across a small defect between left ventricle and right atrium consistent with Gerbode type defect with a small VSD located at the same level (Additional file 3: video S3; Additional file 4: Video S4). The direction of the Doppler signal also leads to the true diagnosis (figure 1C). This defect was the cause of high velocity jet in the right atrium simulating pulmonary arterial hypertension, probably due to the summation of tricuspid regurgitation(TR) and Gerbode defect (figure 1A). Furthermore, the low pulmonic diastolic regurgitation jet velocity was able to unmask this mistake and define this entity. An MRI demonstrated also this connection. A normal lung scan excluded pulmonary embolism.

After 3 weeks of treatment without fever she underwent surgery. Through a right atriotomy, large vegetation in the RA attached to the septal leaflet of tricuspid valve was identified. On removal of the vegetation, except perimembranous septal defect, another defect was found communicating between the left ventricle and right atrium. This defect represented an acquired Gerbode defect and was closed by pledgeted pericardial autolog and running suture. The septal leaflet of tricuspid valve was resected and a tricuspid repair was performed.

After an uneventfully post-operative period, the patient was discharged home in good clinical condition. Echocardiogram demonstrated trivial tricuspid valve regurgitation and no residual shunt.

## Discussion

Septic perforation of the ventricular septum in the course of bacterial endocarditis has been reported rarely in the literature. A Gerbode-type defect (LV-to-RA shunt) due to bacterial endocarditis is even rarer; it has been recorded few times to our knowledge [2,3].

We can only hypothesize about the mechanism of the septal perforation.

Our patient was previously considered a healthy adult female. She had delivered two children and the complete medical examination required at the time had been judged normal. It seems unlikely that her systolic murmur was present and went unnoticed. So, we can also speculate that the probability of a bacterial origin of both defects is high in this case.

The bacterial invasion of any congenital anomaly of the septum is more reasonable. It is possible that her perimembranous ventricular septal defect was congenital in origin and had closed years earlier by any several mechanisms. The mechanism most often implicated is redundant tricuspid tissue that closes the defect when a septal aneururysm forms. Residual septal aneurysm could then perforate secondarily, probably as a result of bacterial invasion from catheter- related bacteremia or nosocomial infection.

It is thought that bacterial infection can affect the subannular region causing rupture of a section of the high membranous septum and the creation of the shunt [3]. The causative organism is usually a Staphylococcus aureus, like in our case [2].

We raise also the question whether minor abnormalities of the ventricular septum, if not overlooked, would require lifelong antibiotic prophylaxis for endocarditis.

## Conclusions

A preoperative diagnosis with color dopler echocardiography of this fistula formation in the setting of infective endocarditis has been reported in the literature, but has been infrequent [3]. However, identification of such communication is often extremely difficult. The high Doppler gradient between the LV and RA on echocardiogram was one of the hallmarks of the Gerbode defect because of the difference between the high left ventricular pressure and the low right atrial pressure [4,5].

Our patient's first echocardiogram was initially misinterpreted as severe pulmonary hypertension, with the Gerbode defect jet not being interpreted correctly.

To avoid overestimating right ventricular systolic pressure by mistaking such a shunt for an eccentric jet of tricuspid regurgitation, it is important to accurately differentiate the two [6]. The anatomic nature of the shunt could be appropriately delineated with the use of meticulous color Doppler echocardiography which is a high sensitive tool for the detection of this rare type of defect.

## Consent

Written informed consent was obtained from the patient for publication of this case report and accompanying images. A copy of the written consent is available for review by Editor-in-Chief of this journal.

## Competing interests

The authors declare that they have no competing interests.

## Authors' contributions

NXH made substantial contribution to perform and analyze the echocardiography patient data and also carried out and drafted the manuscript; EP performed the intervention and revised critically the manuscript; IA made acquisition of MRI and angio-pulmonary scan and revised critically the manuscript; FS provided clinical assistance to the patient;

All authors have given final approval of the version to be published.

## Supplementary Material

Additional file 1 **Video S1 TTE short axis view**. short axis demonstrating a large and highly mobile mass (vegetation) seen in RA. Click here for file

Additional file 2 **Video S2 TTE apical 4-chamber view**. The vegetation seems located just above the tricuspid septal valve without causing obstruction on it. Click here for file

Additional file 3 **Video S3 TEE view**. Four-chamber view showing a subaortic ventricular septal defect and the vegetation with its attachment probably at this level. Click here for file

Additional file 4 **Video S4 Color Doppler TEE**. Systolic flow between the LV and both RV and RA. The color Doppler demonstrates two clear shunts; the one close to the highest portion of the interventricular septum (under the root of the aorta) and the other is high velocity jet from Gerbode type defect mixed with tricuspid regurgitation jet. (LV, left ventricle; RV, right ventricle; RA, right atrium) Click here for file
